# Recommendations for repositories and scientific gateways from a neuroscience perspective

**DOI:** 10.1038/s41597-022-01334-1

**Published:** 2022-05-16

**Authors:** Malin Sandström, Mathew Abrams, Jan G. Bjaalie, Mona Hicks, David N. Kennedy, Arvind Kumar, Jean-Baptiste Poline, Prasun K. Roy, Paul Tiesinga, Thomas Wachtler, Wojtek J. Goscinski

**Affiliations:** 1 INCF Secretariat, Karolinska Institutet, Stockholm, Sweden; 2 Institute of Basic Medical Sciences, University of Oslo, Oslo, Norway; 3 One Mind, Seattle, Washington, US; 4 Department of Psychiatry, University of Massachusetts Chan Medical School, North, Worcester, USA; 5 Div. of Computational Science and Technology, School of Electrical Engineering and Computer Science, KTH Royal Institute of Technology, Stockholm, Sweden; 6 Montreal Neurological Institute, Faculty of Medicine and Health Sciences, McGill University, Montreal, Canada; 7 Computational Neuroscience & Neuroimaging Laboratory, School of Bio-Medical Engineering, Indian Institute of Technology - I.I.T. (B.H.U.), Varanasi, India; 8 Department of Neuroinformatics, Donders Centre for Neuroscience, Faculty of Science, Radboud University, Nijmegen, The Netherlands; 9 Faculty of Biology, Ludwig-Maximilians-Universität München, Planegg-Martinsried, Germany; 10 National Imaging Facility, University of Queensland, St Lucia, Australia; 11 Monash eResearch Centre, Monash University, Clayton, 3800 Australia

**Keywords:** Neuroscience, Research management

## Abstract

Digital services such as repositories and science gateways have become key resources for the neuroscience community, but users often have a hard time orienting themselves in the service landscape to find the best fit for their particular needs. INCF has developed a set of recommendations and associated criteria for choosing or setting up and running a repository or scientific gateway, intended for the neuroscience community, with a FAIR neuroscience perspective.

Digital data repositories play an important role in the archiving, management, analysis and sharing of research data. They provide stable, long-term storage, can improve data quality through active curation, can increase the discoverability and reusability of data through the use of controlled terms and standardized metadata, make it easier to request and transfer data, and help remove or lower barriers to reuse and collaboration. Data shared in repositories is more often cited than data shared by other means, like supplements^1^.

Modern neuroscience datasets are commonly in the gigabyte range, often reach the terabyte level^2,3^, and in some cases the petabyte level^4^. That amount of data is hard to handle without accompanying computational power, data viewing and data analysis capabilities in the same place. We refer to enhanced repositories that provide such resources as scientific gateways, to distinguish them from regular repositories. Throughout, we will use the term “services” to refer jointly to repositories and scientific gateways. Scientific gateways offer computational resources and built-in software resources, sometimes also data visualization, custom data analysis and/or workflow composition. They usually have user accounts and might host data that is only available to logged-in users.

Besides hosting data and providing computational power, repositories and scientific gateways are also important for supporting research reproducibility and replicability; they can preserve data and computational research outcomes that might otherwise be lost or become unfindable over time, and make it realistically possible to redo analyses or computational experiments. Openly available data storage and computational resources also have the possibility to become a driver for increasing diversity and equality in science, as they help counteract differences in access to hardware, tools and resources.

The current repository landscape is quite diverse and varied, and the many different possible choices may thus confuse the intended users. Researchers are often asked to pick the resource that fits them best, but feel they have little guidance to do so^5^.

Therefore, the International Neuroinformatics Coordinating Facility (INCF) has developed selection criteria and associated recommendations (Box 1) for the neuroscience community, with a FAIR^6^ neuroscience perspective, and tried to harmonize them with existing work on criteria for repository selection and best practices from other initiatives, including FAIRsharing^7^, FORCE11^8^ and Coalition of Open Access Repositories (COAR)^9^. We are also taking into account the feedback received in our workshop “Towards neuroscience-centered selection criteria for data repositories and scientific gateways” held on April 26, 2021 at the yearly INCF Assembly^5^.

A detailed version of the recommendations in the form of a checklist is available on the INCF portal (https://www.incf.org/criteria-checklist).

Our aim is two-fold: we want to help neuroscience researchers and students choose good services for their specific use cases; and we want to help service providers make good and future-proof decisions for setup and operations. For this purpose, each section introduces the user perspective first, and then lists recommendations for how service providers can help address this perspective.

The full technical aspects of setting up and running a FAIR service are outside the scope of this comment; we recommend that service providers consult an external resource such as the FAIRSFAIR Basic Framework on FAIRness of services^10^ or the COAR Community Framework for Good Practices in Repositories^8^.

## **Box 1**

Recommendations.

1. Ensure discoverability and transparency in ownership and service usage statistics
2. Clearly communicate access and reuse conditions
3. Consider ethical requirements for authorship transparency and sensitive data
4. Follow best practices for licensing and responsibility
5. Ensure accessibility and interoperability
6. Build capabilities for reproducibility, replicability, reuse
7. Excel in documentation and user support
8. Be transparent in governance and operations
9. Involve community in governance and decision making
10. Be transparent on sustainability - financial and technical

## Ensure discoverability and transparency in ownership and service usage statistics

It is important to ensure that online services are findable and well described. We recommend that services provide a clear and concise description that outlines the resource features, identifies the intended community and states who supports the service. We also recommend that service providers are transparent in communicating their usage data and usage data history as proxies for harder to judge criteria such as community importance, community relevance and impact. Usage statistics methods differ in their approaches and limitations. Service providers need to consider what method provides reliable estimates of the metrics they intend to determine, be careful about privacy, and be transparent in how they obtain their statistics.

We recommend services to register in relevant repository registries, such as Re3data or FAIRSharing, and to consider participating in a certification like Core Trust Seal.

## Clearly communicate conditions for access and reuse

Information on the conditions for access and reuse of a service must be easy to find.

Service providers should clearly state access and deposit conditions and any costs of usage. We recommend that a service clearly and prominently communicates all of the file formats and metadata formats it accepts and uses.

## Consider ethical requirements for authorship transparency and sensitive data

Authorship is a core component of any metadata set. For objects that can be updated, data as well as software, we recommend the service to make it possible to change authorship with any update, and to make the change history of authorship available.

Proof of ethics approval should be required for all data, including data from animal experiments. Services that accept human or otherwise sensitive data should offer the possibility for controlled, verified access, provide information about possible additional requirements, and clearly document how to get access.

## Follow best practices for licensing and responsibility

Clear usage terms and licensing increase the usefulness of shared data.

We recommend service providers to clearly and prominently communicate all access and deposit conditions, and to state a license for downloaded data, software and derivatives. To facilitate reuse, derived data and other downloaded resources should by default have clear licenses. We recommend that services use standard licenses wherever possible (e.g. Creative Commons licenses, https://creativecommons.org/about/cclicenses/) at a clear and appropriate level of granularity. For some types of data, including sensitive data, with conditions not easily covered by licensing, a readable yet sufficiently detailed Data Usage Agreement is required.

Rights and responsibilities of both user and service should be articulated in a clear and transparent manner, with clear terms of use and an end user license or agreement, and for scientific gateways with user accounts also a privacy policy and a code of conduct.

## Ensure accessibility and interoperability

Sharing data in a repository that uses community standards and offers programmatic access will increase its usefulness.

A service can make itself more accessible, interoperable and useful to its target community by using established community standards, for both data and metadata, and community vocabularies. In neuroscience, the BIDS (Brain Imaging Data Structure) format for neuroimaging data^11^, and the NWB (Neurodata Without Borders) format for electrophysiology data^12^ both have strongly facilitated data sharing and collaboration, and the NeuroML markup language^13^ has made it possible to clearly describe, share and reuse computational neuroscience models.

Offering submission in standard formats saves users from having to reformat all their data, makes metadata ingestion easier to support and automate, and results in clear and consistent naming. Broadly available data in community formats will also lower barriers to the development of a surrounding ecosystem of software tools. We also recommend having methods reported in a structured format, a community relevant format if possible.

When community standards are not available, using an applicable general standardization framework is a preferable alternative over designing a new, custom format; this choice increases the likelihood of data and metadata being possible to transform into a future standard format.

Programmatic and command-line access makes modern computational science more productive. We recommend that service providers offer an open, well documented API and/or a command-line interface (CLI) in several community relevant programming languages. Ideally, these interfaces should also be open to community input.

Services should interact with their community and with other community-relevant services to strive for interoperability, consistent access and authorization, and use of community vocabularies.

## Build capabilities for reproducibility, replicability, reuse

Data repositories and scientific gateways have the potential to contribute strongly with technical reproducibility and consistent data quality. Unique identifiers make data easy to find and cite. Structured method reporting and automated metadata verification make data more reliable and reusable.

The use of (machine readable) persistent identifiers (PID) is a core requisite for making research data accessible and fulfilling the FAIR principles. Services should assign PIDs to data descriptions, data and complementary materials (e.g., digital object identifiers (DOI)), software (DOI, Software Heritage ID (SWHID)^14^), authors (open researcher and contributor IDs (ORCID)) and associated research resources (RRIDs^15^). We also recommend that service providers register for an RRID that identifies their infrastructure.

Metadata is critically important to FAIR^6^; it is the backbone of any dataset, and ongoing quality control of metadata is as important as the data. It is vital in ensuring that data can be correctly understood and effectively used and reused.

We recommend services to document and communicate their curation processes for data and metadata. Where possible, higher level curation which links to annotation and other published information material is preferable.

We recommend that methods are reported in a structured, community relevant format, (examples: Structured, Transparent, Accessible Reporting (STAR) Methods, MDAR (Materials Design Analysis Reporting)) and that metadata entry is made easy and automatically or semi-automatically verified. Ideally, methods are also published and citable (using platforms such as protocols.io).

We recommend that key software, such as analysis code, is versioned and documented, and that the versioning history is communicated. Provenance for data, derived data and software should be documented and extractable. We recommend that versioning of both content and authorship is transparently communicated and available for datasets, code, and analysis software.

We recommend services to interact with their community to identify and accommodate various data search behaviours, and to deliver search summaries that make it possible for researchers to judge relevance, accessibility, and reusability of a data collection from the summary.

## Excel in documentation and user support

Sharing data in a user-friendly repository with good documentation and user support will increase its likelihood of reuse. Documentation saves time, resources, and frustration. The importance of good documentation cannot be overstated, ideally documentation is also updated regularly and includes community input. We recommend service providers to have extensive, clear, and readable documentation.

Providing sufficient user support is an essential criterion. Even with good documentation, it can take some time and effort for first time users to orient themselves. We recommend that all service providers have a FAQ with the most common user questions; ideally also a quick start guide. We further recommend that the service providers provide training materials specific to the service; ideally that they also provide or refer users to other relevant training on such issues as FAIR and reproducibility.

We recommend that service providers enable community users to support each other by setting up or utilizing mechanisms such as a user forum or mailing list.

## Be transparent in governance and operations

Users are unlikely to rely on services they do not trust. In research infrastructures trust requires transparency at all levels, from governance to issue handling and communicating updates, outages, and changes. We recommend that service providers document and clearly communicate the governance process, including issue reporting and resolving. Ideally, community users should have influence over governance. Funding sources, or other contributions of value, should be transparently communicated, and any conflicts of interest should be declared.

Services should be operated with information technology best practices, such as communicating outages and changes, establishing a backup and archiving process, having excellent documentation and user support, performing security controls and updates, and allowing for privacy controls if needed.

## Involve community in governance and decision making

Domain-relevant community standards are essential ingredients for the implementation of the FAIR principles. A service’s data and metadata need to meet domain-relevant community standards in order to increase their usability for the intended community. If a community has standards or best practices for data archiving and sharing, services should aim to implement and follow these standards.

It will be hard for services to achieve lasting broad community usefulness and impact without a mechanism for community input and influence; therefore we recommend that all service providers include their intended and actual community in their setup and decision-making process.

## Be transparent on sustainability - financial and technical

Research results are meant to last, and to be possible to revisit and reuse. Therefore, trust and long-term security are important factors in choosing a service for research activities and outputs.

Technical and financial sustainability are both key criteria. Financially, we recommend that service providers transparently communicate current and past grants and other financing. Technically, we recommend that a service provider creates and provides transparent plans for archiving and backup, service closure and data and metadata preservation, and that they support sustainability by using open, established and maintainable technologies in their services.

The sustainability plan should address shutdown and archiving matters such as archiving or data preservation. The service should also state its data preservation policy. We also recommend that the sustainability of the governing body itself is made clear, especially if it is not naturally renewed by elections.

## Conclusion

These recommendations and their associated criteria were developed with the intent to fit repositories as well as scientific gateways. They cover a range of important areas for users selecting digital services, including accessibility, licensing, community responsibility, and the technical and financial sustainability of a service. Transparency and clear communication with users is a common denominator for many of the recommendations.

The recommendations were developed from a neuroscience perspective, but most of them are general and domain agnostic - they apply to data repositories as well as software repositories and science gateways in any scientific field - because they deal primarily with how a service is run and governed.

As a research field, neuroscience has many different communities at very different stages of digital maturity. We hope that our high level recommendations can play a bridging role and look forward to helping communities develop roadmaps towards adopting and implementing them.
